# High-Throughput Incubation and Quantification of Agglutination Assays in a Microfluidic System

**DOI:** 10.3390/genes9060281

**Published:** 2018-06-04

**Authors:** David Castro, David Conchouso, Rimantas Kodzius, Arpys Arevalo, Ian G. Foulds

**Affiliations:** 1 Computer, Electrical and Mathematical Sciences & Engineering Division (CEMSE), King Abdullah University of Science and Technology (KAUST), 4700 KAUST, Thuwal, Jeddah 23955-6900, Saudi Arabia; david.conchouso@kaust.edu.sa (D.C.); rimantas.kodzius@auis.edu.krd and kodzius@envirola.com (R.K.); arpys.arevalo@kaust.edu.sa (A.A.); ian.foulds@ubc.ca (I.G.F.); 2 Mathematics and Natural Sciences Department, The American University of Iraq, Sulaimani, Sulaymaniyah 46001, Iraq; 3 Faculty of Medicine, Ludwig Maximilian University of Munich (LMU), 80539 Munich, Germany; 4 Okanagan Campus, School of Engineering, Faculty of Applied Science, University of British Columbia, 3333 University Way, Kelowna, BC V1V 1V7, Canada

**Keywords:** agglutination assay, lab-on-chip, microfluidics, high-throughput

## Abstract

In this paper, we present a two-phase microfluidic system capable of incubating and quantifying microbead-based agglutination assays. The microfluidic system is based on a simple fabrication solution, which requires only laboratory tubing filled with carrier oil, driven by negative pressure using a syringe pump. We provide a user-friendly interface, in which a pipette is used to insert single droplets of a 1.25-µL volume into a system that is continuously running and therefore works entirely on demand without the need for stopping, resetting or washing the system. These assays are incubated by highly efficient passive mixing with a sample-to-answer time of 2.5 min, a 5–10-fold improvement over traditional agglutination assays. We study system parameters such as channel length, incubation time and flow speed to select optimal assay conditions, using the streptavidin-biotin interaction as a model analyte quantified using optical image processing. We then investigate the effect of changing the concentration of both analyte and microbead concentrations, with a minimum detection limit of 100 ng/mL. The system can be both low- and high-throughput, depending on the rate at which assays are inserted. In our experiments, we were able to easily produce throughputs of 360 assays per hour by simple manual pipetting, which could be increased even further by automation and parallelization. Agglutination assays are a versatile tool, capable of detecting an ever-growing catalog of infectious diseases, proteins and metabolites. A system such as this one is a step towards being able to produce high-throughput microfluidic diagnostic solutions with widespread adoption. The development of analytical techniques in the microfluidic format, such as the one presented in this work, is an important step in being able to continuously monitor the performance and microfluidic outputs of organ-on-chip devices.

## 1. Introduction

There is currently a demand for high-throughput screening of biological samples, particularly in areas such as blood banks, epidemiology, food-borne pathogens and diagnosis of infectious diseases such as human immunodeficiency virus (HIV) and hepatitis [1,2,3]. Agglutination assays, in conjunction with droplet microfluidics and their parallelization, offer an opportunity for research towards meeting this demand [4,5,6].

Immunoagglutination assays are a low-cost, rapid method for the detection of a variety of target analytes, which use the affinity of antigen-antibody complexes to form aggregates of microbeads [1,7]. Functionalized microbeads in an aqueous suspension are mixed with the target analyte, which acts as a binder between them. Consequently, the beads will begin to form aggregates visible to the naked eye indicating a positive result. These tests are typically only qualitative, performed in volumes of 0.2 mL on cardboard cards, incubated in an oscillating mixer for 15–30 min and inspected visually [1,8]. A semi-quantitative assay can be obtained by performing repetitions at a series of dilutions of the analyte and registering the minimum titer that will result in positive agglutination [1]. Because of the large analyte volumes required, these types of assays are not convenient to quantify the output of other microfluidic devices, such as organ-on-chip devices, which we will discuss in the following sections.

These assays were first introduced in the 1950s to diagnose rheumatoid arthritis [9] and have since been developed to detect a very wide variety of analytes and diseases, among them bacterial [10,11,12,13], viral [14,15,16,17] and fungal [18,19], as well as proteins [20,21,22] and DNA [23,24]. They are used in a diverse range of applications, such as veterinary [25], food safety [26,27], rapid clinical and field diagnosis [22]. An important field of research has been the diagnosis of infectious agents, particularly HIV [16], which has been one of the greatest motivators for the development of novel immunoassays [3].

In general, the performance of immunoassays can be enhanced in two areas: the first is by improving the efficiency of the formation of the antigen-antibody complex, and the second is by improving the detection of these complexes [3]. Microfluidics can offer potential advantages in both areas, as it can provide novel platforms for incubation and promote the binding of the antigen-antibody, as well as novel assay detection and quantification methods. Additionally, microfluidics offers the now well-known advantages of high-throughput, low reagent consumption and higher degrees of sample control. Consequently, in the past decade, there has been an increasing interest in integrating agglutination assays and microfluidic devices [28,29]. A variety of detection methods in microfluidics has been demonstrated such as qualitative visual inspection [30,31], flow cytometry and light scattering [32,33,34], resistive pulse sensing [35], fluorescence [31,36], visible light microscopy [37] and optical image processing [38,39,40].

Most work involving agglutination in microfluidic systems, which we will now briefly present, operate in single-phase, meaning that only the aqueous suspension of particles is flowed continuously through the length of the microchannel where the assay is processed. Therefore, these devices typically rely on introducing previously-incubated assays into the microchannel [33], or in diffusional mixing with careful selection of surfactants [37]. Other devices propose more complex solutions that allow the performance of multi-step assays with incubation done within a single device. In these examples, the microbeads are first introduced and immobilized at the channel walls, by using beads modified with a temperature-sensitive polymer [41,42] or magnetic actuation [36,43]. An analyte is then flowed past the beads, which will aggregate, after which the beads are released from the channel walls to proceed to assay quantification. Although these methods allow for more complex multi-step protocols, the sample-to-answer time is over 30 min for each assay and can only be performed one at a time, resulting in lower throughput.

One solution to quantify some of these assays is to utilize a mechanical barrier to capture the particles, such as using a microfilter [32] or features and chambers designed on the device [30,31]. Although these methods allow the assays to be readily evaluated, they require either thorough washing after each use, or for the device to be disposable, which makes them therefore inherently single-use and low-throughput.

Although single-phase systems are simple to design and implement, they have two well-known issues especially relevant for agglutination microfluidic devices [44,45,46]. The first one, known as Taylor dispersion, is a consequence of the parabolic laminar flow profile typically found in microfluidic systems [47]. Since flow rates are higher towards the center of the microchannel and slower towards the walls, this makes solutes and particles have different residence and incubation times inside a microchannel. Secondly, since the phase containing the assay is in direct contact with the microchannel, particles and reagents can deposit on the channel walls and lead to cross-contamination or fouling [37,48].

Conversely, in two-phase flow, two immiscible fluids are flowed through microchannels, where they are forced to interact at a junction point to create discrete volumes of one phase (the disperse phase) surrounded by the other (the continuous phase). If the disperse phase fills the entire cross-section of the microchannel, it is called a plug, and if it does not, then it is called a droplet [45].

It is well documented that plugs in two-phase flow do not present Taylor diffusion, but they instead have an internal recirculation and produce a rapid mixing of reagents within [49,50], which can be used to enhance the mixing and incubation of an assay. A full recirculation inside the plug will occur as long as it is allowed to travel several times its own length [51,52]. There is an added complexity in the case where the aqueous phase contains a suspension of particles. Depending on the flow conditions, these particles can exhibit a range of recirculation patterns, from full recirculation across the entire plug, to concentrating the particles in small regions at the ends of the plug [53,54]. Selecting the flow parameters carefully, particles will be carried and recirculated efficiently by the flow patterns inside the plug [54].

Cross-contamination is also minimized in two-phase flow [48,55], since the aqueous phase is not directly in contact with the microchannel walls, particularly when using hydrophobic channel materials.

Despite these advantages, there are few examples in the literature of agglutination in two-phase microfluidics, which we will briefly present. An interesting approach taken by some researchers has been to immobilize a droplet containing an assay that is floating on carrier oil and studying the incubation dynamics [56,57]. Rastogi et al. [56] immobilized the droplet using dielectrophoresis and studied the dynamics caused by induced evaporation, and Sivashankar et al. [57] suspended the droplet at the interface of two immiscible fluids and monitored in real-time the incubation enhanced by mechanical agitation. While these approaches provide quantitative measurements, their long incubation times (>30 min) and sequential nature provide no improvement over the incubation times of traditional card-based tests.

An example using two-phase agglutination in traditional microchannels is the work of Kline et al. [38], who demonstrated agglutination assays for blood typing and *Staphylococcus aureus* in 40-nL plugs produced by a droplet generator. These plugs flow through a microchannel with complex serpentine geometries that enhance their mixing and incubation. During operation, this system can produce hundreds of assays per hour at varying dilutions; however, each assay contains the analyte from the same source. If different sources are to be analyzed, such as the case of a sample from a different patient, the system must be stopped, and the generator reconfigured to be able to proceed. Teste et al. [39] demonstrated a fully-automated platform in which 100-nL plugs containing a model analyte (streptavidin-biotinylated phosphatase alkaline) were quantified at a rate of 300 assays per hour. These plugs were automatically generated in batches from a microtiter plate by a pipetting robot. This system however required the assays to be previously incubated, as well as synchronous magnetic actuation, which increased its complexity and lowered its throughput.

Although for soft lithography, Polydimethylsiloxane (PDMS) has become a staple material in this field, simpler and alternative fabrication methods are worth exploring. Low-complexity fabrication, such as the use of laboratory tubing and connectors, is a simple and interesting approach, particularly when the application requires round cross-sections or relatively longer channels [40,58,59,60,61,62,63]. A wide variety of applications has been demonstrated using laboratory tubing as microchannels, such as crystallization [58,59,60], generation and storage of droplet libraries [61,62] and complex multi-step assays [63], among others.

In this work, we address the issues described above and present a low-cost, plug-based platform that integrates incubation and quantification of microbead-based assays, building on a preliminary platform previously introduced by our group [40].

We use incubation during plug flow to decrease the incubation time to 2.5 min and provide throughput of 360 assays per hour by simple hand pipetting. Based on our flow rates and assay size, as is elaborated in the discussion, with automation of the assay introduction, the system with a single microchannel could handle a theoretical maximum of 3600 assays per hour. If parallelized under a single detector, it could theoretically provide >25,000 samples per hour. We study different system parameters, such as flow rate, microchannel length and their relation to assay incubation, to optimize assay performance. The microchannel is based on simple laboratory tubing that involves minimal fabrication complexity and provides highly efficient mixing of the assays employing passive phenomena and flow parameters, with no active elements required. Quantification of the assays is done optically with a simple camera and image processing. Although this technique is less sensitive than others such as laser-scattering flow cytometry [33], optical imaging has a wide field of view and can cover several microchannels in a single frame, which is conducive to parallelization. It also has the advantage of being easier to implement, as illustrated by its wide adoption [64,65,66,67].

Finally, one of the issues in the current state of microfluidics and its attempts to produce successful commercial applications is the interaction between the micro-scale device and the macro-scale operators [4] and user-friendliness [68,69]. In our setup, we have emphasized the importance of user-friendliness, easily interfacing with a standard laboratory pipette to introduce the assays into the microfluidic system.

## 2. Materials and Methods

### 2.1. System Description

**Figure 1:**
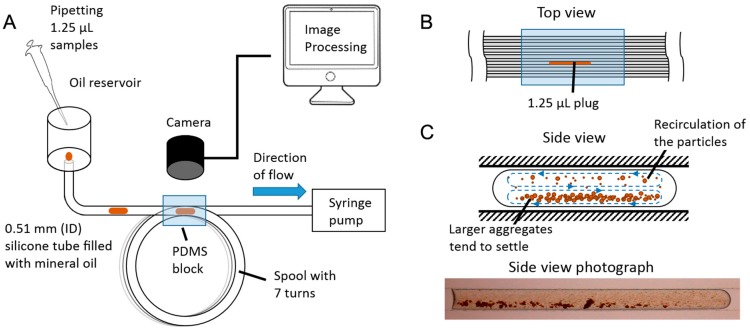
(**A**) Schematic of the continuous high-throughput microfluidic system. The assays are introduced in 1.25-µL volumes into a 0.51-mm internal diameter silicone tube of 2 m in length through an open oil reservoir. The entire tube is filled with mineral oil, which is continuously drawn from the reservoir by a syringe pump connected at the opposite end. The tube is coiled around a spool, and seven observation windows have been created by casting a polydimethylsiloxane (PDMS) block, which provides refractive index matching with the material of the tube. The length of each turn in the coil is 30 cm. A camera has been positioned above the observation windows, enabling us to capture the passage of the assay through the tube at regular intervals. (**B**) Top view of the PDMS block, illustrating a plug traveling through an observation window. The field of view of the camera is 9.7 × 9.7 mm. (**C**) Side view diagram and photograph of an assay, indicating recirculation patterns and microbead aggregate behavior.

The general setup is shown in Figure 1. It consists of a fluid-handling component that performs the incubation of the assays and a camera setup with image processing performed post-hoc that takes care of the quantification of the assay.

The fluid-handling component consists of a 2-m commercially available silicone tube, with an internal diameter of 0.51 mm (silastic). This choice was made because it allows us to easily have a circular microfluidic channel of longer lengths (>1 m) with minimum fabrication requirements. Conventional microfluidic devices made from PDMS soft lithography require the creation of a master mold, casting, baking and bonding, and typically only produce channels with a rectangular cross-section. A channel with a rectangular cross-section will have a higher hydraulic resistance than one with a circular cross-section of similar dimensions and will therefore require a higher pressure to drive the same amount of flow [70,71].

The tube is filled with light mineral oil (Sigma Aldrich, St. Louis, MO, USA). One end of the tube is connected to a syringe pump that continuously draws the liquids at a fixed speed, and the other open end is connected to an oil reservoir, which can hold approximately 4 mL of oil and is replenished as the level drops. A pipette is used to insert the assays into the tube, which will then flow through its entire length and will be discarded once they reach the syringe pump.

The tube was coiled around a spool in turns of 30 cm to be able to store it compactly. A digital camera (5D Mark II) (Canon, Tokyo, Japan) with a macro lens (MP-E 65 mm 1–5× macro) (Canon) was positioned above the spool to be able to observe the passage of the assays at regular intervals. A PDMS block was cast around the tubes to minimize the optical effect of the silicone tube walls by refractive index matching, effectively creating an observation window that encompasses seven turns of the spool (Figure 1B). A white LED lamp was placed underneath this window to provide uniform and repeatable lighting.

**Figure 2:**
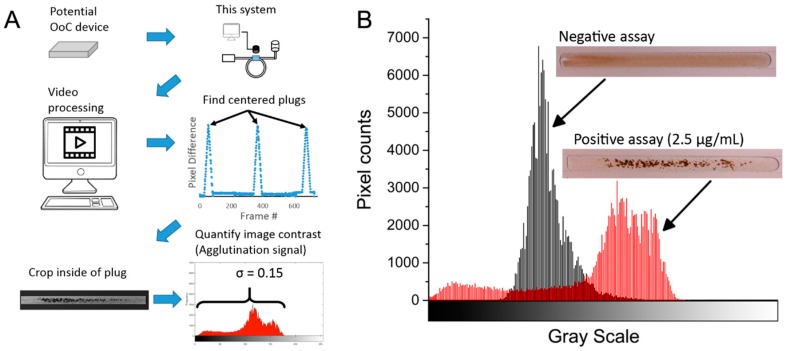
(**A**) Example of plug quantification and workflow. The image processing algorithm detects changes in the field of view of a video and identifies the frame containing a complete and centered image of a plug. A region of interest of the interior of the plug is cropped, and the standard deviation of its grayscale values is obtained. (**B**) Example histograms of a negative (microbeads + simple bovine serum albumin (BSA)) and a positive test (microbeads + biotinylated BSA, 2.5 µg/mL in the example shown). In a negative test, the uniform suspension of microbeads is seen as a low contrast region with a narrow grayscale distribution. Conversely, a positive test will have dark aggregates of microbeads, resulting in higher contrast and a wider histogram. OoC:Organ-on-Chip; σ: standard deviation.

The camera records video of the passing plugs, with a resolution of 1920 × 1080 pixels at a rate of 30 frames per second. This output from the camera is then processed in MATLAB (MathWorks Inc., Natick, MA, USA), where we implemented an algorithm that identifies plugs and measures the degree of agglutination within them, illustrated in Figure 2A.

First, to detect the frames that contain a complete plug, each frame of the video is subtracted pixel-wise from a reference frame of empty microchannels. The frames that produce the highest difference from the reference frame indicate a fully-centered plug, whose internal area is then cropped and converted to grayscale. This cropped image contains the information of the degree of agglutination in the assay (specific aggregation of microbeads) in the form of image contrast. To better understand this, a visual representation of contrast is seen in the image’s histogram (Figure 2B). An assay with no agglutination (low concentrations of analyte) will have an even coloring throughout the plug and therefore a narrow distribution seen in its histogram (low contrast). An assay with a high degree of agglutination, on the other hand, will have a wide distribution in color, with darker areas corresponding to microbead clumps, as well as lighter areas where no beads are present (high contrast). The spread of this histogram can be quantified by its standard deviation (σ), which correlates with the degree of agglutination and therefore with the concentration of the analyte in the assay. The original image is in grayscale value units (0–255), which are then normalized to 0–1. After calculating the image’s standard deviation, the range is now 0–0.5. This single number is a representation of the contrast of the image, which for brevity we will refer to as the “agglutination signal” expressed in arbitrary units (a.u.).

Incubation of the assay occurs passively as the plug travels through the length of the microchannel. In laminar flow, drag against the walls produces a parabolic velocity gradient across the cross-section of the microchannel, being maximum at the center and approaching zero at the walls. In single-phase flow, this can result in Taylor dispersion [47], causing a variation of residence times of the reagents inside a channel. In segmented flow, the parabolic flow profile from the frame of reference of the plug produces a recirculation pattern (Figure 1C). This recirculation pattern moves forwards in the direction of the flow at the center of the channel and reverses direction at approximately 70% of the channel width [54]. A full recirculation inside the plug will occur as long as it is allowed to travel several times its own length [51,52]. Since our plugs travel several hundred times their own length, this ensures that this condition is met.

### 2.2 Assay Protocol

For our experiments, we selected biotin-streptavidin, a specific protein-protein interaction, as a model agglutination assay, consisting of streptavidin microbeads and biotinylated bovine serum albumin (BSA). The biotin-streptavidin interaction is used widely for its specificity and high binding strength [72] and has been used extensively in microfluidics research to model immunoagglutination assays [24,33,35,36,39,43].

The microbeads used were polystyrene Dynabeads M-270 (Thermo Fisher Scientific, Waltham, MA, USA), with a diameter of 2.8 µm suspended in Phosphate-Buffered Saline (PBS), at a concentration of approximately 6–7 × 10^8^ beads/mL. The biotinylated BSA (Thermo Fisher Scientific), used as the model analyte, comes in lyophilized powder form and was restored with PBS, and both components were further diluted as required by the experiments using the same buffer. As a negative test, we used simple BSA at similar concentrations. The stability of the droplet as it travels through the tube is of great importance, so Tween 20 nonionic surfactant was added to the PBS buffer of the microbead suspension at a concentration of 0.1% by weight. The microbeads were magnetically separated, the supernatant removed and replaced with this buffer with surfactant, according to the manufacturer’s recommendations. This wash protocol also helps minimize the presence of free streptavidin in the suspension. This concentration of surfactant was optimal to produce stable single plugs, preventing them from breaking into smaller droplets by the shear forces of the flow as they travel through the channel. We require an intact 1.25-µL droplet to be able to quantify the assay; therefore, plug stability is necessary. Using a nonionic surfactant additionally acts as a blocker of the hydrophobic surfaces of the microbeads, which reduces nonspecific agglutination of the microbeads [7,73].

To perform a single assay, the microbeads were vortexed and then sonicated for three min to ensure a uniform suspension and avoid nonspecific aggregates, and 4 µL were pipetted into a 0.2-mL well. Next, 4 µL of analyte were added into the well to form a bead-analyte mixture, from which 1.25 µL were immediately drawn and inserted into the microfluidic system. This was done in a user-friendly way, as all that was required was to submerge the pipette tip into the open oil reservoir (Figure 1) and release the assay droplet at the opening of the silicone tube. Since the pump was continuously running, the assay droplet could be easily introduced into the silicone tube at any point in time, in which incubation would commence as described in the previous section.

## 3. Results and Discussion

**Figure 3:**
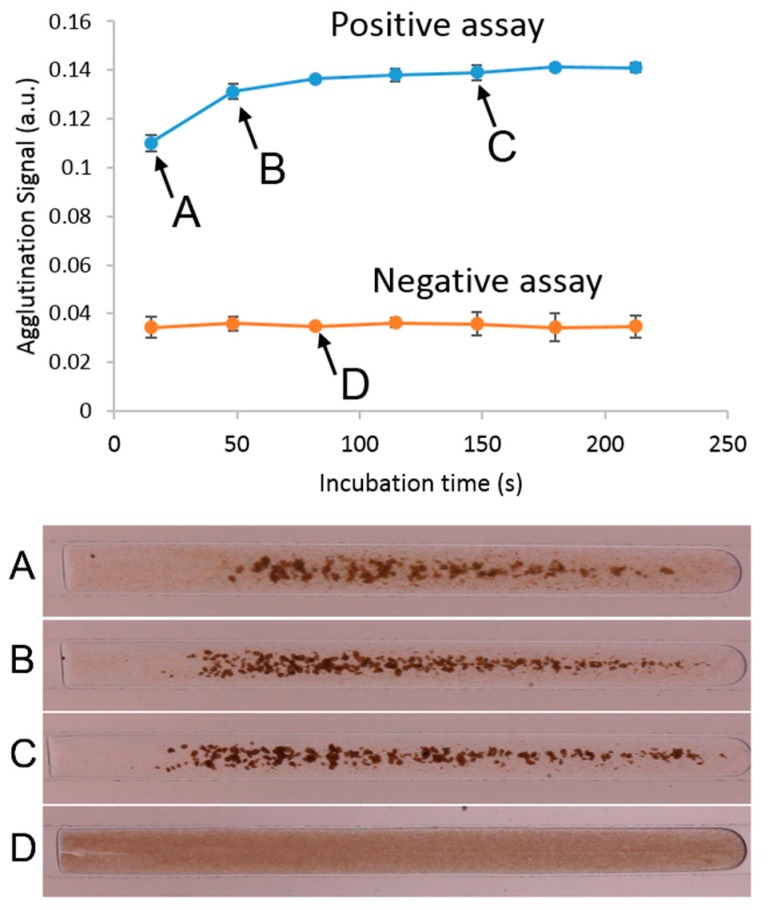
An example of a typical agglutination assay in this system, as the plug travels through the entire length of the tube. Each measurement was taken at 30-cm increments along the channel, located at the PDMS inspection point. This graph shows the progression of the agglutination, with insets showing the frames captured at each window. Points (**A**–**C**) show a positive agglutination test (2.5 µg/mL biotinylated BSA), and point (**D**) shows a negative test.

We monitored the progression of the assay as it travelled through the system to ensure incubation reached a steady state [74]. Reaching equilibrium, or a steady state, is desirable, as this gives the assay more stability even when having small differences in conditions between tests [75,76]. Figure 3 shows an example of a single experiment, using an analyte concentration of 2.5 µg/mL at a speed of 175 µL/min. As seen in the photographs, the assay progressively forms aggregates that are visible as areas of higher contrast (Figure 3A–C). We plot this agglutination signal as a function of incubation time and observe an increase in the signal until the fifth window (approximately 150 s). At this point, the agglutination begins to show saturation, indicating that sufficient incubation has occurred and that the tube is of sufficient length to complete the assay. It can also be seen that a negative test, consisting of only BSA, will not show any change as the droplet travels through the windows, as no agglutination is taking place, and remains as an even dispersion of microbeads (Figure 3D).

As previously discussed, cross-contamination is minimized in two-phase flow [48,55], particularly when using hydrophobic channel materials. We routinely measured negative test assays run through the system and found no detectable cross-contamination between consecutive assays as expected. This characteristic is consistent with the literature, as some researchers [63,77] have shown similar plug-based assays in which microbeads are magnetically manipulated and transported from one aqueous plug to another, with no signs of cross-contamination.

An interesting phenomenon we observed, shown in Figure 1C, was that as the microbeads agglutinate and form larger aggregates, they would tend to settle at the bottom of the plug. The drag force *D* that a spherical particle experiences in a fluid is given by Stokes’ law of resistance [78]:

(1) D=πμrU,

where *µ* is the dynamic viscosity of the fluid, r is the radius of the particle and *U* is the relative velocity between the fluid and the particle. Equating this drag force with the effective weight of a spherical particle 4*πr*^3^*g(ρ_p_ − ρ_f_)/*3 and solving for U gives an expression for sedimentation velocity (*U_s_*):

(2) $$U_{s}=\frac{2}{9}\frac{\left(\rho_{p}-\rho_{f}\right)r^{2}g}{\mu },$$

where *ρ_p_* is the density of the particle, *ρ_f_* is the density of the fluid and *g* is the acceleration of gravity.

With all other parameters being equal, Equation (2) indicates that an increase in a particle’s radius, which is an approximation to the formation of a larger microbead aggregates, will result in a considerable increase of its settling speed; see Table 1. This implies therefore that larger aggregates will tend to settle at the bottom half of the plug under the influence of gravity, forming a bed of slower circulating aggregates. This behavior mimics the effect of immobilizing the microbeads at the wall of the channel to allow the antibody to flow over them and bind to the antigen sites, which other researchers have achieved actively using a temperature-sensitive polymer [41] or magnetic actuation [43]. We produce a similar effect in our system by entirely passive phenomena and have a highly efficient mixing of the beads and analyte. This mixing results from the combined effect of the internal circulation patterns of the plug and the settling of large aggregates, which make unbound beads and analyte flow past the larger aggregates that settle at the underside of the plug. As a result of this phenomenon, the agglutination rate is increased in our system.

**Figure 4:**
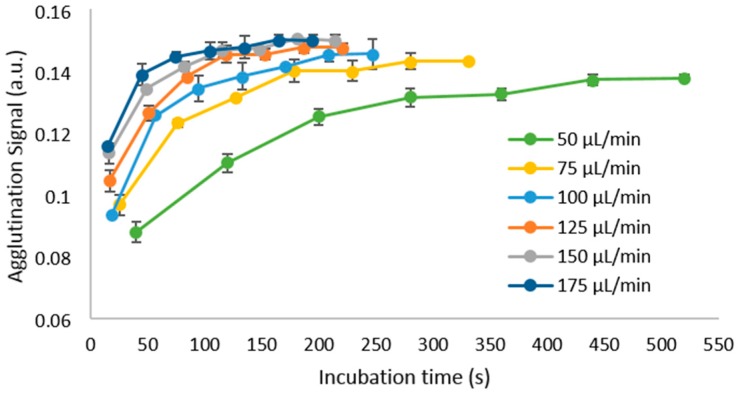
Effect of increasing the flow rate of the system, using a concentration of 2.5 µg/mL biotinylated BSA, as the plugs travel through the tube. Each data point represents the average of 15 measurements, and the error bars show the standard deviation between them.

To optimize the system parameters, we first investigated the influence of the flow rate of the carrier oil on the incubation of assays. We determined that a reasonable range of flow rates to test would be between 50 µL/min and 175 µL/min. Due to the use of a flexible tube and a negative pressure, the channel walls begin to collapse at higher pressures, and therefore, values higher than 175 µL/min no longer produce higher actual flow rates within the channel. Assays with a fixed concentration of 2.5 µg/mL biotinylated BSA were performed at that range, and the results are shown in Figure 4.

Reaching equilibrium, or a steady state, is desirable as this gives the assay more stability even when having small differences in conditions between tests [75,76]. At 50 µL/min, the mixing rate will be lowest, which results in a lower magnitude of the measured agglutination signal, while simultaneously taking more time to reach a steady state. Increasing flow rate shortens the time required to saturate the agglutination signal, reaching a maximum at 175 µL/min. We also found that at flow rates higher than the ones shown, the final agglutination signal begins to decrease, as the higher shear forces within the plug can overcome the binding forces of the assay and begin to separate the aggregates of beads.

From these results, we have selected 175 µL/min as the optimal flow rate for the system at which subsequent experiments will be conducted, as it can provide adequate mixing and assay saturation in the shortest incubation time of the different speeds tested. In these conditions, we obtain a sample-to-answer time of approximately 2.5 min (150 s), with a throughput limited to the rate at which the assays can be pipetted into the reservoir.

During our experiments, we were able to introduce assays at a rate of approximately 360 assays per hour by simple manual pipetting. By tracking the order or timing at which each assay was inserted, it became simple to match and identify their corresponding measurement. Although this rate constitutes a considerable improvement over traditional agglutination assays, which typically take 15–30 min (2–4 assays per hour), it is far from the maximum that the system would be capable of handling. To calculate the theoretical throughput limit, we measured the amount of time required for an assay to pass by the field of view of the camera as 0.5 s (15 frames) at the optimal flow rate to our system (175 µL/min). Assuming a train of assays in which a plug’s length is left as a separation between each assay, we could achieve a theoretical maximum throughput of ~3600 samples per hour. If instead of manual pipetting, the assay introduction was done by automated pipetting or by interfacing the input of the system with a tubing-based library of analytes [5,61,62], throughput numbers closer to this maximum limit could be achieved. This number could be further increased by parallelization [79,80] of independent microchannels under the same camera. In our system, we used a wide field of view to monitor the same microchannel at seven points in regular intervals (one for each turn of the spool). Instead of measuring one channel at seven points, we could measure seven independent channels at a single point each, where the incubation has reached its steady state. With this modification, the current optical setup could theoretically have in its field of view seven independent channels, each with its own train of assays, providing > 25,000 samples per hour, a seven-fold increase.

**Figure 5:**
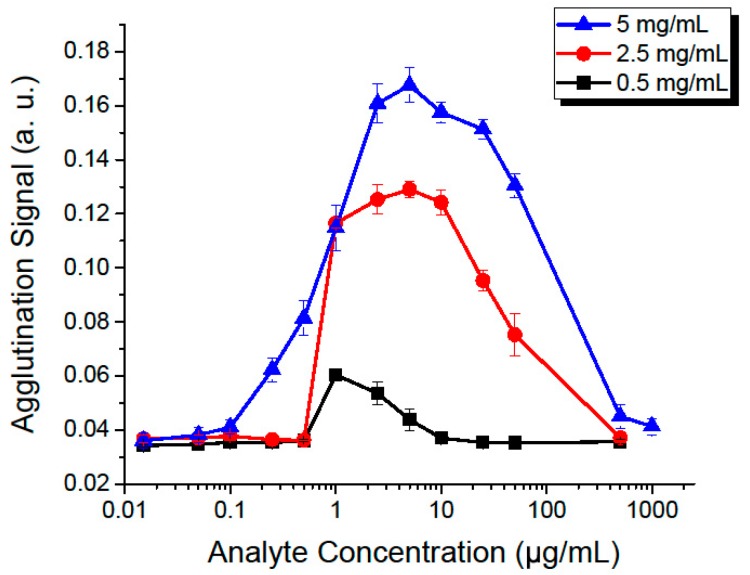
Effect of increasing the microbead and analyte concentrations, at a fixed flow rate of 175 µL/min, with an incubation time of 150 s. Each point represents repetitions of five experiments, with error bars showing the standard deviation between them. Each curve was made with a different concentration of microbeads, from 0.5–5 mg/mL.

After selecting the optimal flow rate (175 µL/min) and incubation time (150 s, occurring at the sixth observation window), we then proceeded to study the effect of the concentrations of both the analyte and microbeads, as shown in Figure 5.

As concentrations increase, we measure a higher agglutination signal until it reaches a maximum and then begins to decrease. This phenomenon is known as the prozone or Hook effect [11,81], a false-negative that is caused by saturating the microbeads with the analyte. If all sites of two neighboring microbeads are saturated, then they will be unable to bind to each other, effectively reducing the degree of agglutination. The prozone effect appears at higher analyte concentrations when the microbead concentration is increased. This is to be expected, as the higher microbead count requires more analyte molecules to saturate. A serial dilution can be performed to allow measurements that would be unmeasurable otherwise, thus extending the usable range of the test.

A negative assay produces an agglutination signal of 0.036 with a standard deviation of 0.0018. Defining our limit of detection (LOD) as three standard deviations above the negative reference (agglutination signal of 0.0414), we determined that the minimum concentration that we can detect is 100 ng/mL. This value is a clinically-relevant detection level for applications such as C-reactive protein (CRP) [20], *S. aureus* [26,82], immunoglobulin G (IgG) [21] and M (IgM) [22]. When using a different agglutination assay, for example with a weaker binding interaction or lower affinity, a new dose curve similar to the one shown in Figure 5 could be quickly made and the system parameters adjusted accordingly. It is also important to note that the sensitivity of the assay can be fine-tuned by modifying the assay protocol and adjusting the added volumes of the functionalized microbead suspension and of the analyte. In our experiments, we maintained a 1:1 proportion of added volumes of microbeads and of analyte. Increasing the analyte proportion tends to shift the dose curves towards the left, effectively lowering the detection threshold as this increases the number of analyte molecules per microbead within the assay.

A field of research that has been attracting much attention in recent years is comprised of the so-called “Organ-on-Chip” (OoC) devices [83,84,85]. They combine the fabrication techniques developed by the field of microfluidics together with 3D cell culture [86], to produce novel platforms that more closely resemble in vivo conditions [83]. These platforms are providing new insights into the areas of drug delivery [87], drug screening [88] and cell characterization [89]. One of the challenges facing this field is the need for the development of analytical techniques suitable for the small dimensions and output volumes that are typically found in OoC devices [84]. Depending on the scale and parallelized cultures per device, typical outputs of OoC devices are a few microliters per hour (1–5 μL/h) [90,91], tens to hundreds of microliters per hour (24–200 μL/h) [92,93] or a few milliliters per hour in cases of higher parallelization of cultures per device (0.6–1.5 mL/h) [94,95]. The mismatch between OoC readout techniques and clinical diagnostic tests [96] can be addressed by the development of novel analysis systems in microfluidic formats, compatible with their low volume outputs, which is the subject of this work.

As previously discussed, we have focused our system on high-throughput, and therefore, our camera setup uses a wider field of view to be able to span the width of several microchannels. A natural consequence of this lower magnification is that individual microbeads and small aggregates cannot be as clearly resolved, resulting in lower sensitivity. This detection limit could be improved by sacrificing the capability for parallelization and using higher magnification lenses. It could be improved upon even further by completely changing the detection method, for example with laser scattering-based quantification.

## 4. Conclusions

In this paper, we have demonstrated a straightforward and user-friendly microfluidic device capable of incubating and quantifying plug-based agglutination assays of 1.25-µL volumes, a 100× smaller consumption of reagents as compared to traditional assays. The low volumes of both analyte and reagents required by this system are particularly interesting for the ever-growing field of organ-on-chip. As previously discussed, typical outputs of OoC devices range from a few microliters per hour (1–5 μL/h) [90,91], to a few milliliters per hour in cases of higher parallelization (0.6–1.5 mL/h) [94,95]. These outputs are inherently incompatible with traditional quantification techniques and assays, as it could take several hours to fulfill the analyte requirements to be able to perform a single test. Our system represents work towards addressing this mismatch, as it requires only a few microliters of analyte per test. Additionally, as agglutination tests can be designed to detect a wide variety of molecules and biomarkers, this work represents a versatile tool that could be adapted to monitor different OoC devices. Future work could include the direct integration of this system with an OoC device, to produce continuous on-chip quantification of its output.

This system can continuously handle assays at both high and low-throughputs, interfacing with a standard laboratory pipette on demand, processing > 360 assays per hour by manual pipetting. This number could be increased to an ideal limit of 3600 assays per hour in a single microchannel if a different method of sample introduction were used, such as automated pipetting or interfacing with a pre-existing droplet library. This number could be increased even further (25,000 assays per hour) by the parallelization of several channels, which can be imaged within the same field of view of the camera.

We used the biotin-streptavidin interaction as a model analyte to characterize our system and explored the system parameters that affect the incubation rate of the assay, selecting 175 µL/min and of 160 cm as the optimal flow rate and microchannel length, respectively. With these settings, we can provide a sample-to-answer time of 2.5 min, which constitutes a 5–10× reduction of the incubation time of standard agglutination assays. Incubation occurs efficiently within the plugs as they circulate through the microchannel, aided by the passive internal recirculation patterns. We obtained dose curves for increasing concentrations of analyte and microbead suspension, with a detection limit of 100 ng/mL, which is adequate for many agglutination assay applications, such as immunoglobulin G and M, C-reactive protein and *S. aureus*. The system could be quickly adapted to other similar microbead-based assays, by generating its corresponding dose or concentration curves and adjusting the system parameters accordingly. Future work could attempt to reduce this detection limit and increase sensitivity, by modifying the quantification method with turbidity or nephelometry.

User-friendly interfaces such as the one we have presented are an attempt to close the gap between lab-on-a-chip solutions with biologists and healthcare professionals who have no previous experience working with microfluidics.

With an increase in world population and the desire for improved healthcare, novel methods for quantification and detection of diseases and various biomarkers at high-throughput are needed. Immunoagglutination assays are available for an ever-growing range of applications, and those that deal with large-scale issues such as drinking water quality, food-borne pathogens or infectious diseases such as HIV are under continuous development. Agglutination assays are simple and reliable tests, and enhancing their performance thanks to the advantages that microfluidics can provide is a topic that is worth exploring. This work provides researchers with a simple and low-cost tool that can provide on-demand continuous quantification of agglutination assays and is easily extended to very high-throughputs without major modifications.

## Figures and Tables

**Table 1:** Different particle settling velocities (*U_s_*) as a function of the particle diameter, from Equation (2). Here, we consider *ρ_p_* = 1.6 g/cm^3^ (polystyrene), *ρ_f_* = 1 g/cm^3^ and *µ* = 1.002 mPa s (water).

Particle Diameter (µm)	*U_s_* (m/s)	*U_s_* (mm/day)
1	3.26 × 10^−7^	28.2
2.8	2.56 × 10^−6^	221
5	8.6 × 10^−6^	705
10	3.26 × 10^−5^	2.82 × 10^3^
50	8.16 × 10^−4^	7.05 × 10^4^
100	3.26 × 10^−3^	2.82 × 10^5^
500	8.16 × 10^−2^	7.05 × 10^6^
